# Insights into community profiles, environmental influence, and assembly mechanisms of oyster-associated bacteriome from Yueqing Bay, China via absolute quantitation by metabarcoding

**DOI:** 10.3389/fmicb.2026.1752237

**Published:** 2026-04-10

**Authors:** Huai Lin, Xin Li, Linhao Chen, Tingting Zhang, Fengxia Yang, Yi Luo

**Affiliations:** 1 State Key Laboratory of Water Pollution Control and Green Resource Recycling, School of the Environment, Nanjing University, Nanjing, China; 2 Agro-Environment Protection Institute, Ministry of Agriculture and Rural Affairs, Tianjin, China

**Keywords:** 16S rRNA, assembly mechanism, bacteriome, environment, oysters

## Abstract

**Background:**

Oyster populations create new habitats, maintain productivity, improve water quality by removing excess nutrients in coastal environments, and provide a source of animal protein. More importantly, their associated bacteriome is likely a primary contributor to these ecological functions. However, the community profiles, assembly mechanisms of oyster-associated bacteria, and their interaction with the surrounding environment remain poorly understood.

**Methods:**

In this study, 24 oysters were collected from four typical oyster farming sites from Yueqing Bay, China, and subjected to spike-in absolute high-throughput 16S rRNA amplicon sequencing and subsequent bioinformatic analysis.

**Results and discussion:**

The results show that the pan-bacteriome of oysters from Yueqing was dominated by Proteobacteria at the phylum and *Vibrio* at the genus level, which were the most prevalent and abundant bacteria. Moreover, the structure of the oyster bacteriome from different sampling sites revealed significant geographic differences. These variations were found to be significantly correlated with the physicochemical properties, such as salinity, total nitrogen, ammoniacal nitrogen (NH_4_^+^-N), nitrate nitrogen (NO_3_^−^-N), etc. Importantly, analyses of the neutral community model (NCM) and null model revealed that stochasticity dominated the assembly of the oyster bacteriome. Such ecological processes may contribute to maintaining the diversity and stability of the oyster bacteriome.

**Conclusion:**

Collectively, the findings of this study enhance our understanding of the patterns of farmed oyster bacteriome across different environmental conditions and their underlying assembly mechanisms, thereby providing essential insights for evaluating coastal ecosystem health and valuable guidance for sustainable oyster farming practices.

## 1 Introduction

Oysters are bivalve mollusks that feed by filtering suspended organic particles, microalgae, phytoplankton, and bacteria. They play an important role in the functioning of coastal marine ecosystems. For example, oyster populations contribute to conservation and sustainable uses of the seas by creating new habitats, maintaining productivity, cleaning water through removal of suspended particulates and excess nutrients (Newell et al., 2005; Kellogg et al., 2014), and providing a source of animal protein at a low greenhouse gas cost (Ray et al., 2019). Especially, due to the high nutritional quality (high level of high-quality protein as well as minerals such as zinc and selenium) (Zhang et al., 2025), oysters are widely consumed as food in the world (Willer and Aldridge, 2020). The global oyster market has grown significantly over the past two decades, with particularly robust expansion in China. They were widely farmed in China Bay along the 18,000-km coastline, from north to south, accounting for 80% production of the world (Botta et al., 2020; Sun et al., 2023). Consequently, oysters are ecologically and economically important for the sustainable development of the health of the coastal area in China.

Yueqing Bay is a semi-enclosed bay located on the northern side of the Oujiang River estuary in southeastern Zhejiang Province, China. It covers an area of approximately 469 km^2^ and a coastline of 220 km. This region is known as the “golden bay” and is one of the important marine aquaculture bases in China, where oysters are largely farmed. Historically, oyster farming has been a traditional family practice that contributes to the income of local fishermen. The sustainability of oyster farming in Yueqing Bay is tightly linked to the stability of its farming ecosystems, which are jointly shaped by biotic and abiotic physicochemical parameters. This connection highlights the relevance of studying oyster-associated bacteriomes, as they are not only integral to the bay’s ecosystem function but also play critical roles in oyster growth and health. For example, their associated bacteriome participates in biogeochemical cycling through the remineralization of organic matter and the transformation of carbon (C), nitrogen (N), and sulfur (S) between different chemical forms (Unzueta-Martínez et al., 2022; Lei et al., 2025). Previous studies have reported that the host habitat is the major determinant of the microbiome in marine species (Kim et al., 2021), and these microbiomes play an important role in their growth by providing complementary functional resources to the host, such as nutrient metabolism, immune system modulation, and pathogen resistance (Roeselers et al., 2011; Geraylou et al., 2012; Trabal Fernández et al., 2014). These findings suggest that the bacteriome of oysters from different species and different locations varies and that external environment factors, such as salinity, temperature, pH, etc., may exert a significant influence on the microbiome of live marine food organisms (Sullam et al., 2012; Kim et al., 2021; Unzueta-Martínez et al., 2022). For example, previous research has analyzed the gut bacteriome composition of oysters such as *Crassostrea virginica*, *Crassostrea corteziensis*, *Crassostrea gigas*, and *Crassostrea sikamea* (Trabal Fernández et al., 2014; Pierce et al., 2019; Lei et al., 2025) and found that Proteobacteria was consistently identified as the most abundant phylum across different oyster species (Trabal Fernández et al., 2014). However, the bacteriome composition of oyster may vary with geographic locations, which is consistent with previous studies demonstrating geographic variation in the structure of oysters associated bacterial communities (Trabal Fernández et al., 2014; Lei et al., 2025). Therefore, despite previous studies exhibiting the structure of the oyster-associated bacteriome, knowledge of bacterial community profiles in Yueqing Bay and its interaction with the region’s specific environmental conditions remains limited.

A major goal of microbial community ecology is to understand the forces that structure community composition. The assembly mechanisms of ecological communities are fundamentally governed by niche-based and neutral theories (Riddley et al., 2025). According to these frameworks, natural communities are shaped by a combination of niche-mediated and stochastic factors, integrating both neutral and niche dynamics that operate across distinct spatiotemporal scales (Wang et al., 2013; Wu et al., 2021; Li et al., 2025). For example, Liu et al. found that stochastic factors controlled a small percentage of microbiota entering oysters, providing plasticity that enables them to maintain microbial homeostasis, while the majority of oyster microbiota are residents governed by deterministic factors (Liu et al., 2024). Liu et al. found that redbelly Tilapia-associated gut microbiomes are dominated by stochastic processes (Liu et al., 2025). Specifically, the ecological niche theory emphasizes deterministic processes driven by environmental selection. These processes include abiotic factors, such as nutrient availability, and biotic interactions, such as mutualism and competition, which play critical roles in community assembly (Riddley et al., 2025). Consistent with this theory, studies have identified salinity as a key determinant of oyster-associated bacterial community composition and functions (Lei et al., 2025). They found that increasing salinity significantly influenced the abundance of dominant bacterial genera such as *Vibrio*, *Pseudomonas*, and *Shewanella*, thereby altering the composition of bacterial communities. In contrast, the neutral theory posits that stochastic processes, such as homogenizing dispersal, dispersal limitation, and ecological drift, exert an important influence on community assembly. Notably, environmental changes can trigger substantial shifts in the relative dominance of these processes: bacterial communities may transition from being primarily structured by stochasticity to being dominated by determinism, or vice versa. At larger spatial scales, the influence of stochastic processes tends to outweigh that of deterministic processes, whereas at smaller scales, deterministic processes may be more dominant (Yan et al., 2017). Therefore, natural communities are more often shaped by the combined effects of stochastic and deterministic processes (Li et al., 2025; Riddley et al., 2025). However, the mechanisms underlying bacterial community assembly in oysters remain largely unknown. Distinguishing these processes and quantifying their relative contribution is critical for assessing the resilience and vulnerability of the oyster bacteriome. Specifically, such study can clarify how the oyster bacteriome responds to environmental disturbance and pathogen infection (Li et al., 2017).

High-throughput sequencing of the 16S rRNA gene is now widely used to characterize complex bacterial communities in depth and has greatly advanced our understanding of oyster-associated communities. Although this approach offers high taxonomic resolution, it provides only the relative abundance of the taxon. Moreover, interpretation of microbial community differences among samples based solely on relative abundances can, however, be misleading because fluctuations in the absolute abundance of one species may cause an apparent change in the measured (relative) abundance of other species (Tsilimigras and Fodor, 2016; Props et al., 2017). To address this limitation, spike-in standard-based high-throughput 16S rRNA gene amplicon sequencing has been developed for the absolute quantification of the bacteriome (Tourlousse et al., 2016; Props et al., 2017; Ji et al., 2019). These standards consist of 12 sequences with full-length 16S rRNA genes containing artificial variable regions with negligible identity to known nucleotide sequences, allowing unambiguous identification of spike-in sequences in 16S-seq read data from any microbiome sample. This advanced approach retains the phylogenetic resolution of 16S rRNA sequencing, while providing comprehensive quality control and absolute quantification of oyster bacteriome in Yueqing Bay, thereby enabling more reliable exploration of how the local environment specifically interacts with and shapes the composition of these bacterial communities.

In this study, we sampled oysters from Yueqing Bay, China, and characterized their associated bacteriome using a quantitative high-throughput 16S rRNA gene amplicon sequencing approach. The objectives of this study were to investigate the community profiles of oyster bacteriome, analyze the interaction between bacteriome and environmental factors, and uncover the underlying mechanisms of bacterial community assembly. Our findings are expected to enrich the studies on marine microbial community ecology and provide guidance for sustainable oyster aquaculture.

## 2 Materials and methods

### 2.1 Sample collection and environmental factor measurement

We collected 24 adult oysters (*Crassostrea gigas*, the most widely farmed oyster species in China) for bacteriome analysis from four sampling sites of Yueqing Bay of Zhejiang Province, China, in October 2024. These sites were sampled based on the two main criteria: all sites are natural oyster habitats where the same oyster species is currently farmed; the sites span a gradient of key abiotic factors known to influence both oyster and microbial community, such as salinity (ranging from 7.36 to 25.60 parts per thousand), dissolved oxygen (ranging from 7.52 to 9.10 mg/L), and total nitrogen (ranging from 0.80 to 1.23 mg/L). These biotic and abiotic factors may allow us to test the assembly mechanism of the oyster-associated bacteriome. These oysters are randomly selected with a shell height of 6–12 cm and an age of 7–12 months after the postlarvae, according to a previous study (Trabal Fernández et al., 2014), to minimize the influence of growth stage on the bacterial community. After collection, all oyster samples were promptly kept in ice and immediately transferred to the lab. The detailed geographical coordinates of four sampling sites, such as D3, D4, D5, and D6, are provided in Table 1. Environmental parameters of the farming waters were measured *in situ* using a DZ-C aquaculture water quality detector (Qingdao Juchuang Environmental Protection Group Co., LTD). The parameters assessed included water temperature, pH, salinity, ammonium nitrogen (NH_4_^+^-N), nitrate nitrogen (NO_3_^−^-N), total organic carbon (TOC), total dissolved solids (TDS), and dissolved oxygen (DO). The results of these parameter measurements are provided in Supplementary Table S1.

**Table 1:** Detailed information on sampling sites and corresponding environmental factors.

Site	Altitude	Longitude	NH4^+-^N (mg/L)	TN (mg/L)	NO3^—^N (mg/L)	TP (mg/L)	TOC (mg/L)	Salinity (ppt)	Temperature (°C)	TDS (mg/L)	DO (mg/L)	pH	Samples
D3	121.09311	28.28112	0.46 ± 0.21	1.05 ± 1.20	0.57 ± 0.06	0.06 ± 0.02	1.14 ± 0.12	7.36 ± 0.2	21.9 ± 1.5	7.2 ± 1.2	7.68 ± 2.1	7.92 ± 0.5	D3NO1, D3NO2, D3NO3, D3PO1, D3PO2, D3PO3, D3PO4
D4	121.09743	28.28549	0.70 ± 0.17	1.23 ± 0.5	0.53 ± 0.08	0.06 ± 0.02	1.54 ± 0.26	8.73 ± 0.11	21.4 ± 3.9	8.4 ± 1.9	7.52 ± 0.02	7.82 ± 1.5	D4NO1, D4NO2, D4NO3, D4PO1, D4PO4, D4PO5
D5	121.1240	28.2733	0.27 ± 0.10	0.80 ± 0.12	0.54 ± 0.20	0.04 ± 0.13	1.20 ± 0.32	17.44 ± 0.25	22.8 ± 0.05	17.03 ± 4.5	8.31 ± 3.45	7.52 ± 1.65	D5NO1, D5NO2, D5NO3, D5NO4, D5NO5
D6	121.1240	28.2733	0.40 ± 0.01	0.86 ± 0.42	0.45 ± 0.10	0.05 ± 0.01	1.28 ± 0.57	25.6 ± 1.3	20.5 ± 2.5	24.7 ± 3.3	9.1 ± 1.5	7.96 ± 3.3	D6NO1, D6NO2, D6NO3, D6PO1, D6PO2, D6PN3

### 2.2 DNA extraction and absolute quantification 16S rRNA amplicon sequencing

Prior to microbial DNA extraction, all oysters were opened after surface disinfection with 75% ethanol to eliminate external bacterial contaminants. For bacteriome analysis in this study, internal oyster tissues were used as a whole without distinguishing oyster gill, mantle, or gut tissues. Microbial DNA was extracted using E. Z. N. A. Stool DNA Kit (Omega Biotek, GA, U. S.). The quality and concentration of the extracted DNA were assessed and quantified by 1.0% agarose gel electrophoresis and a Nanodrop 2000 spectrophotometer (Thermo Scientific, United States).

Absolute quantification of bacteria 16S rRNA amplicon sequencing was conducted by Majorbio Bio-Pharm Technology Co., Ltd. (Shanghai, China). To enable absolute quantification, 12 different spike-in sequences (with four different concentrations: 10^3^, 10^4^, 10^5^, and 10^6^ copies of internal standards) were added to the sample DNA pools. The spike-in standards sequences were used as previously designed and can be found in GeneBank under accession numbers LC140931-42 (Tourlousse et al., 2016). These spike-in sequences contained conserved regions identical to those of selected natural 16S rRNA genes, while their artificial variable regions shared negligible nucleotide sequence identity with public databases. This design allowed the spike-in sequences to function as internal standards and facilitate consistent absolute quantification across all samples.

The hypervariable regions V3–V4 of the bacterial 16S rRNA gene were amplified with primer pair 338F (5’-ACTCCTACGGGAGGCAGCAG-3′) and 806R (5’GGACTACHVGGGTWTCTAAT-3′) (Klindworth et al., 2013; Lin et al., 2020) with a T100 Thermal Cycler PCR thermocycler (Bio-Rad, USA). The PCR reaction mix (total volume 20 μL) contained 4 μL 5 × Fast Pfu buffer, 2 μL 2.5 mM dNTPs, 0.8 μL of each 5 μM primer, 0.4 μL of Fast Pfu polymerase, 10 ng of template DNA, and ddH_2_O to bring the volume to 20 μL. The thermal cycling conditions were as follows: initial denaturation at 95 °C for 3 min, followed by 27 cycles of denaturing at 95 °C for 30 s, annealing at 55 °C for 30 s, extension at 72 °C for 45 s, and a single extension at 72 °C for 10 min, and completion at 12 °C. PCR products were analyzed by 2% agarose gel electrophoresis and purified using the PCR Clean-Up Kit (YuHua, Shanghai, China) according to the manufacturer’s instructions and quantified using a Qubit 4.0 fluorometer (Thermo Fisher Scientific, USA).

### 2.3 High-throughput sequencing and analysis

Purified amplicons were pooled in equimolar amounts and subjected to paired-end sequencing on the Illumina NextSeq2000 platform. Raw FASTQ files were de-multiplexed using an in-house Perl script, followed by quality-filtering with fastp version 0.19.6 (Chen et al., 2018) and merged by FLASH version 1.2.7 (Magoč and Salzberg, 2011). Optimized sequences were clustered into operational units (OTUs) using UPARSE 7.1 (Edgar, 2013) with a 97% sequence similarity cutoff. For each OTU, the most abundant sequence was selected as the representative sequence. OTU representative sequences assigned to 12 spike-in sequences (with 100% identity cutoff) were filtered out, and the remaining reads were counted. A standard curve was generated for each sample adjusted by spike-in DNA copy number standard curve, and based on this curve, the quantitative abundance of each OTU in the corresponding sample was determined. Taxonomic annotation of each OTU representative sequence was performed using the RDP classifier version 2.2 (Wang et al., 2007) against the 16S rRNA gene database (e.g., Silva v138) using a confidence threshold of 0.7. The raw sequencing reads were deposited in the NCBI under BioProject PRJNA1359352.

### 2.4 Statistical analysis

Based on the OTUs dataset, *α* and *β* diversity indices were calculated using the R packages “vegan” and “ggplot2.” The similarity of the microbial communities among different samples was visualized via non-metric multidimensional scaling (NMDS) based on Bray–Curtis dissimilarity and using the PERMANOVA test to assess the percentage of variation explained, along with its statistical significance using the Vegan v2.5–3 package. Linear discriminant analysis (LDA) effect size (LEfSe) was performed to identify bacterial taxa with significantly differential abundance among sites (LDA > 2, *p* < 0.05) (Segata et al., 2011). To investigate the effects of environmental factors on oyster bacteriome structure, redundancy analysis (RDA)/canonical correspondence analysis (CCA) was performed using the Vegan V2.5–3 package. The differences in bacterial composition in oysters across sampling sites are presented as mean ± SD. Comparisons of two sites were conducted using a Student’s t-test using SPSS 19.0, with a *p*-value < 0.05 considered statistically significant. The assembly processes of the community were assessed using Sloan’s neutral community model (NCM) (Sloan et al., 2006) and a null model based on a two-step framework detailed in Stegen et al. (2013) to quantify the relative contribution of stochastic and deterministic processes at the individual taxa/lineages level. For the null model analysis, the *β*-nearest taxon index (βNTI) and Raup-Crick Matrix (RCbray) were used to identify the ecological mechanism driving variations in phylogenetic and taxonomic diversity (Shi et al., 2023). βNTI was used to indicate the extent to which compositional changes between two communities are driven by deterministic or stochastic factors. Specifically, a βNTI > 2 indicates that coexisting taxa are more closely related than expected by change (phylogenetic clustering), or a βNTI < −2 indicates that coexisting taxa are more distantly related than expected by change (phylogenetic overdispersion). βNTI values within the ranges mentioned above imply that communities were assembled by deterministic processes, such as heterogeneous and homogeneous selection. When βNTI values fall within the interval of −2 ≦ βNTI ≦ 2, community assembly is governed by stochastic processes. RCbray is calculated from |βNT| < 2, which means that the replacement of a group of communities is by dispersal limitation, homogenizing dispersal, and undominated control of the processes. RCbary > 0.95, RCbray < −0.95, and −0.95 ≦ RCbary ≦0.95 indicate dispersal limitation, homogenizing dispersal, and undominated (such as weak selection, weak dispersal, diversification, and drift), respectively (Dini-Andreote et al., 2015).

## 3 Results

### 3.1 Pan-bacteriome of oysters

**Figure 1:**
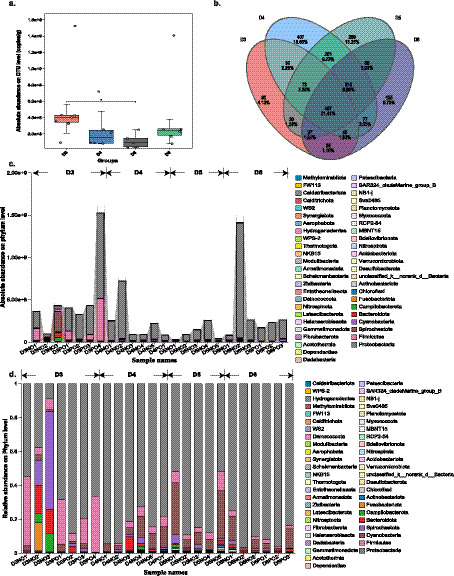
Pan-bacteriome of oysters from different sampling sites in Yueqing Bay, China. **(a)** Absolute abundance of bacteria in oysters. **(b)** Venn diagram of bacteriome in oyster from different sites at the OTU level. **(c)** Absolute abundance of bacteria of oysters at the phylum level. D3, D4, D5, and D6 represent the sampling sites, and NO or PO with a different number were the oysters’ code. **(d)** Absolute abundance of bacteria of oysters at the genus level.

Using spike-in 16S rRNA amplicon sequence-based absolute quantification, we analyzed the bacteriome of 24 oysters collected from four sampling sites in Yueqing Bay, China. Overall, the total detected bacteria were classified into 48 phyla, 859 genus, and 1,268 species. The bacterial load of the oysters ranged from 2.8E+07 to 1.52E+09 copies/g, with an average of 3.32E+08 ± 3.77E+08 copies/g (Figure 1a; Supplementary Table S2). Among the four sampling sites, oyster from site D3 showed the highest bacteria load of 5.09E+08 ± 4.28E+08 copies/g (Figure 1a). In contrast, oysters from site D5 had the lowest bacterial load, at 1.11E+08 ± 8.2E+07 copies/g (Figure 1a).

Oysters from different sampling sites shared limited bacteria. Out of all OTUs detected, only 21.41% (467) were found across all samples (Figure 1b). The most abundant cored OTUs were OTU244 (accounting for 30.26% of co-shared OTU), OTU247 (accounting for 17.33% of co-shared OTU), and OTU243 (accounting for 10.52% of co-shared OTU) (Supplementary Figure S1). These three OTUs were annotated as *Vibrio campbellii*, *Citrobacter freundii,* and *unclassified Acinetobacter*, all of which belong to the phylum Proteobacteria. Among the four sampling sites, oysters from the D4 site showed the highest unique OTUs (407), accounting for 18.66% of the total OTUs (Figure 1b), followed by oysters from site D5, which harbored 289 unique OTUs (Figure 1b). At the phylum level, the five most abundant phyla found in the oyster bacteriome were Proteobacteria, Firmicutes, Spirochaetota, Cyanobacteria, and Bacteroidota (Figure 1c). Thereinto, Proteobacteria was the most prevalent and abundant phylum, with its relative proportion ranging from 8.75 to 96.93% and an average of 75.47 ± 20.91% (Figure 1d). At the genus level, *Vibrio* and *Citrobacter* were the most abundant genera across all oyster samples (Supplementary Figures S2a,b). For *Vibrio*, the relative proportion in oysters ranged from 0 to 91.68%, with an average bacterial load of 1.26E+08 ± 2.51E+08 copies/g and an average relative proportion of 43.50% ± 29.44% (Supplementary Figures S2a,b). Oysters from the site D6 had the highest *Vibrio* load, reaching 1.29E+09 copies/g (Supplementary Figure S2a). For *Citrobacter*, the relative proportion in oysters ranged from 0 to 84.85%, with an average load of 8.27E+08 ± 1.70E+08 copies/g and an average relative proportion of 17.125% ± 22.02% (Supplementary Figures S2a,b). The highest *Citrobacter* load was observed in oysters from site D3 at 2.05E+08 copies/g (Supplementary Figures S2a,b). Together, these results characterize the bacteriome of oysters from Yueqing Bay, China.

### 3.2 The oyster bacteriome exhibits site-specific geographic variation

**Figure 2:**
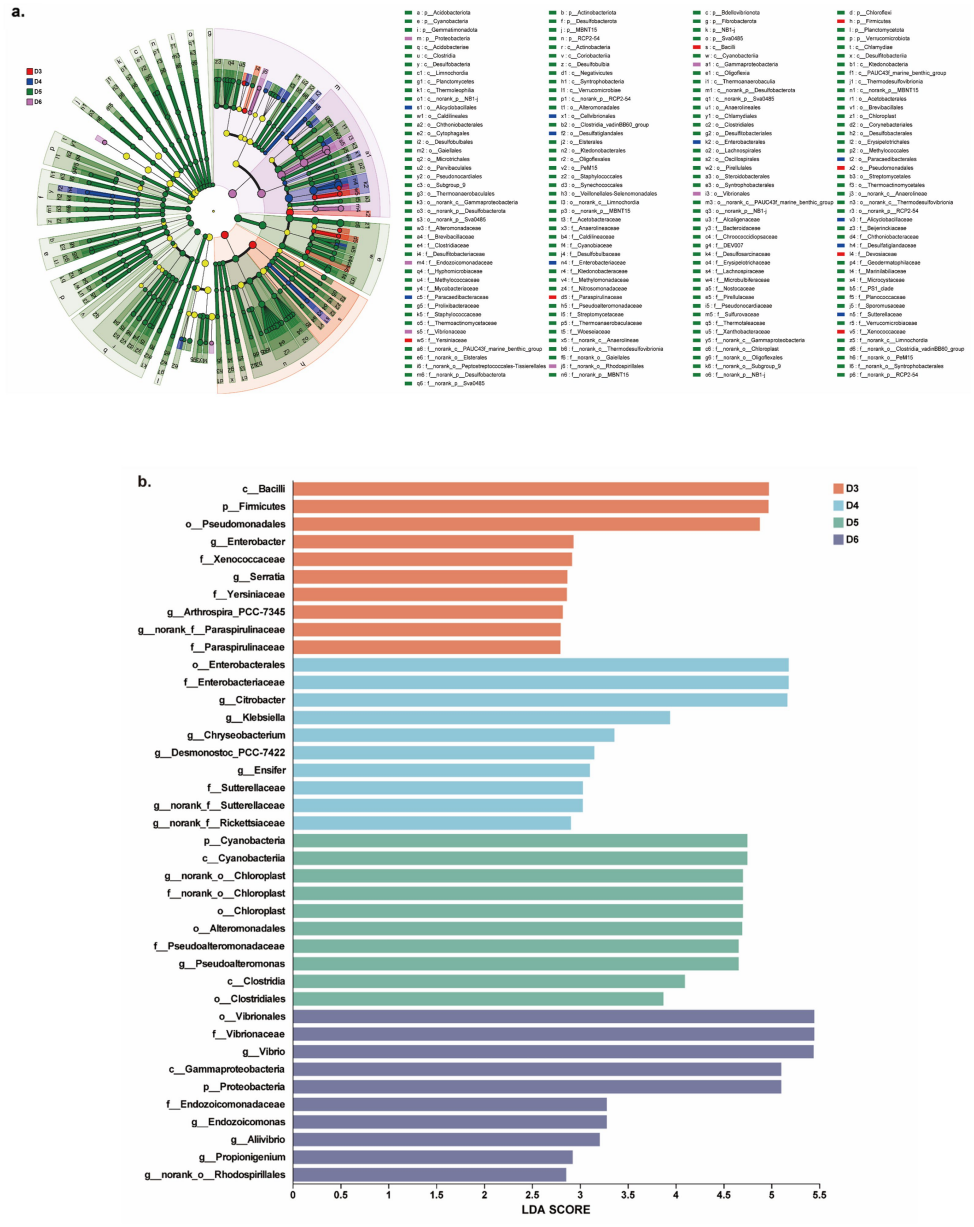
Taxonomic difference in oysters among different sampling sites revealed by LEfSe. **(a)** Cladogram of differential bacteria at different taxonomies. This cladogram only exhibits differential bacteria at the family level. **(b)** LDA scores of differential bacteria from different sites. The blot shows only the top 10 differential bacteria in each group.

**Figure 3:**
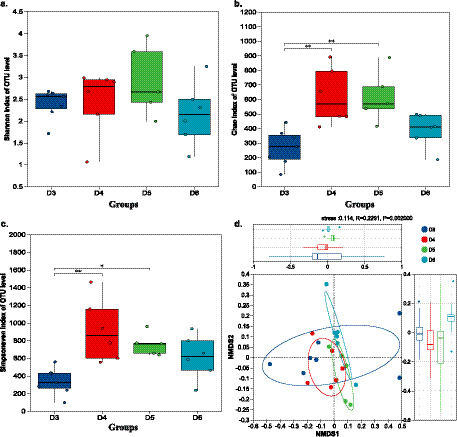
*α* and *β* diversity of the oyster bacterial community. **(a–c)** Shannon, Chao, and Simpson are even of α diversity representative bacterial community diversity, richness, and evenness. **(d)** β diversity of oyster bacteriome.

The structure of the bacteriome differed among oysters from different sampling sites. LEfSe analysis identified abundant marker bacteria belonging to 20 phyla and 111 genera from different sites (Figure 2a; Supplementary Table S3). Among them, oysters from site D5 harbored the highest number of marker bacteria at various taxonomic levels compared to oysters from the other sites. The top 10 marker bacteria ranked by LDA score in each site are shown in Figure 2b. For example, *Enterobacter* and *Serratia* were enriched in oysters from site D3, *Citrobacter* and *Klebsiella* were enriched in those from site D4, *Vibrio* and *Endozoicomonas* were enriched in site D6, and *Pseudoalteromonas* was enriched in site D5. These results suggested that the bacterial composition of oysters from different sites varied. Consistent with this finding, the *α* diversity of the oyster bacteriome also shows significant difference. Although the diversity of the oyster bacteriome did not exhibit significant variation across sites (based on Shannon index, Figure 3a), their richness and evenness showed significant differences (Figures 3b,c, *p* < 0.05). Specifically, oysters from site D4 had the highest community richness and evenness, while those from site D3 had the lowest. Corresponding to the differences in community diversity of the oyster bacteriome across different sites, their structure exhibited a significant difference in *β* diversity (Figure 3d, *p* < 0.05, PERMANOVA). Collectively, these findings suggested that the bacteriome of the oyster exhibited habitat-specific profiles, with distinct characteristics associated with each sampling site in Yueqing Bay.

### 3.3 Influence of environmental factors on oyster-associated bacteriome

**Figure 4:**
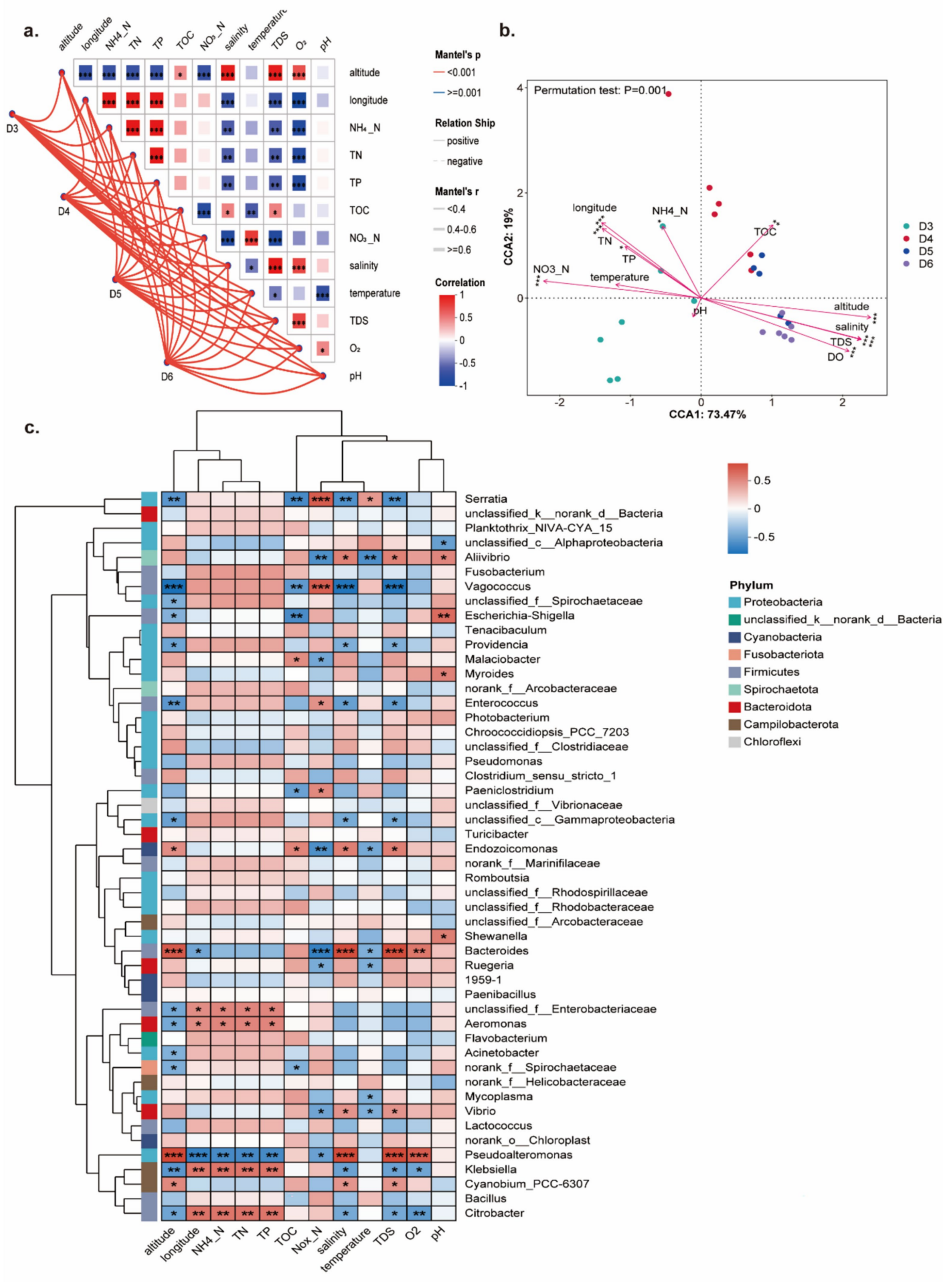
The influence of environmental factors on oyster bacterial community. **(a,b)** Mantel test and CCA between environmental factors and the oyster bacterial community. **(c)** Correlation of environmental factors and bacteria at the species level. *p < 0.05, **p < 0.01 and ***p < 0.001.

The bacteriome of oysters from different sites was significantly impacted by environmental factors, as revealed by the Mantel test (Figure 4a). As we know, geographic patterns drive the heterogeneity of environmental factors. From the Mantel test, we further verified that geographic coordinates, especially altitude, were significantly correlated to all measured environmental factors (Figure 4a). Moreover, these differences in environmental factors exerted a significant influence on the bacteriome of oysters. This finding was validated by CCA, which revealed that all detected environmental factors, except temperature and pH, significantly affected the oyster bacteriome, such as NH_4_^+^-N, NO_3_^−^-N, TN, TP, salinity, TDS, and DO (Figure 4b). The correlation between environmental factors and bacterial genera further supported these results (Figure 4c). Specifically, genera including *Pseudoalteromonas*, *Klebsiella*, and *Citrobacter* exhibited stronger correlation with environmental factors than other genera (Figure 4c), suggesting they play an important role in adapting to environmental variations. *Vibrio* species show a strong correlation with salinity and temperature (Figure 4c), indicating they may exhibit specific responses to these environmental factors. Together, these results suggested that the environmental factors play a role in shaping the oyster bacteriome in Yueqing Bay.

### 3.4 Stochastic process shapes the geographic pattern of bacterial communities of oysters

**Figure 5:**
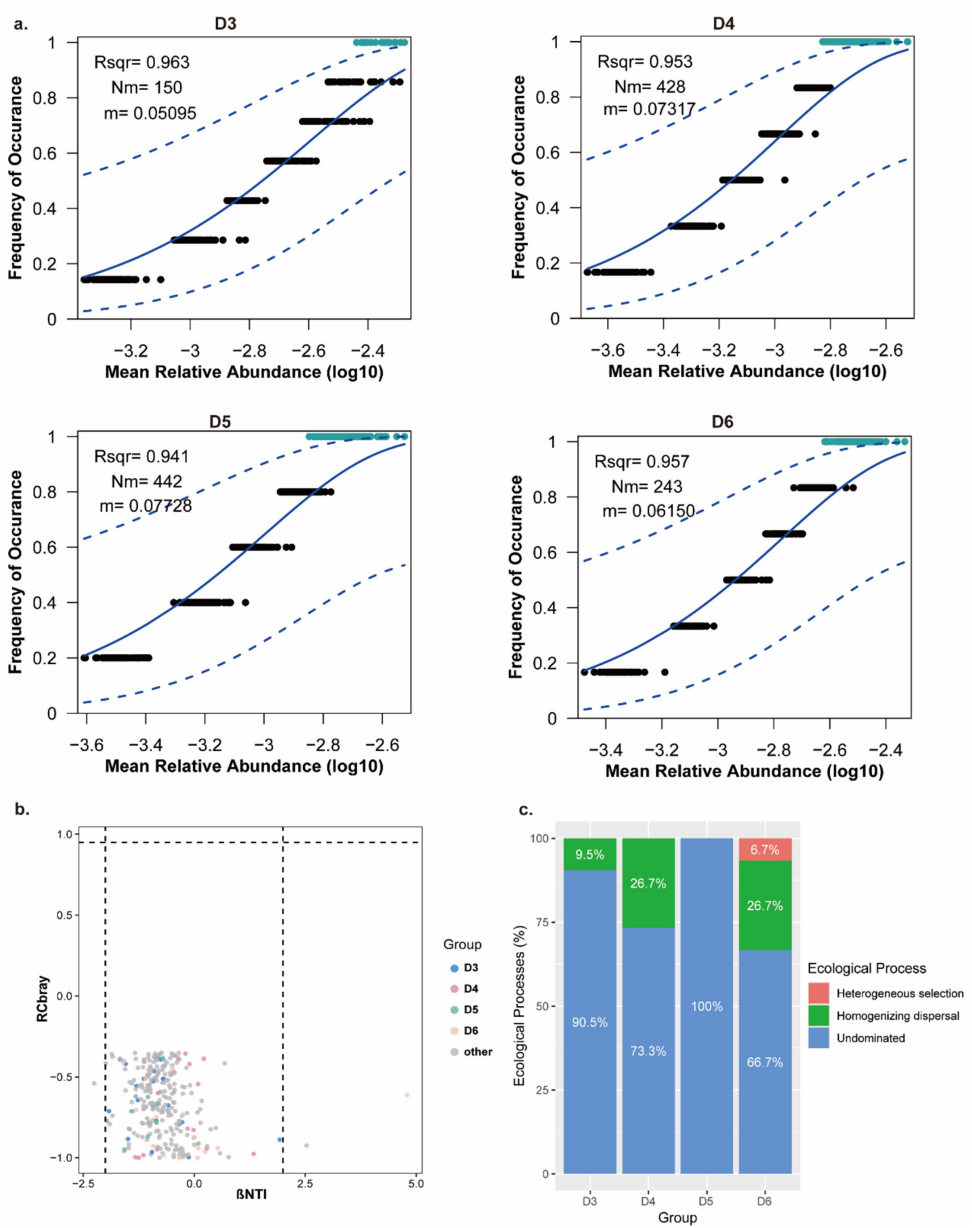
Assembly mechanism of oyster bacteriome based on NCM and null model. **(a)** NCM of oysters from different sampling sites. **(b)** βNTI and RCbray value of the oyster bacterial community from different sites. **(c)** Ecological process contribution to the oyster bacteriome from different sites.

NCM results revealed that the bacteriome of oysters from different sites was dominated by a stochastic process, where the coefficient of determination (R^2^) for these communities was >0.9, which suggested a strong fit of the NCM to the observed data (Figure 5a). Additionally, the migration rates (m) of the four oyster bacterial communities were low (ranging from 0.05 to 0.08), indicating strong dispersal limitation across all groups. These findings were consistent with the results of the null model analysis, which further supported the role of stochastic processes in bacterial community assembly. The null model exhibited that all bacterial communities from oysters had |βNTI| < 2 and |RCbary| < 0.95—conditions indicating that undominated stochastic processes, such as weak selection, weak dispersal, diversification, and ecological drift, exerted the greatest impact (Figure 5b; Supplementary Figure S3). This undominated process accounts for 66.7 to 100% of the ecological process shaping the oyster bacteriome (Figure 5C). Additionally, other processes, such as homogenizing dispersal, also influenced the oyster bacteriome, accounting for 0 to 26.7% of the ecological process (Figure 5C). Taken together, these results demonstrate that stochastic processes are the primary drivers of bacteriome assembly in oysters from Yueqing Bay.

## 4 Discussion

The bacteriome of oysters is influenced by both biotic and abiotic factors and is involved in the health of their host and the coastal ecosystem (Ray and Fulweiler, 2021; Unzueta-Martínez et al., 2022; Lei et al., 2025). Therefore, characterizing the profiles of the oyster bacteriome, along with its underlying driving factor and assembly mechanisms, is essential to understanding their ecology. Here, by using spike-in standard-based high-throughput 16S rRNA amplicon sequencing and analysis, we found that the oyster bacteriome in Yueqing Bay, China, exhibits geographically distinct patterns and is dominated by stochastic assembly processes, with its structure influenced by local environmental factors.

Proteobacteria were the most abundant bacterial phylum in oysters, which is consistent with previous studies (Prapaiwong et al., 2009; Trabal et al., 2012; Trabal Fernández et al., 2014; Yu et al., 2021; Lei et al., 2025). This, owing to the fact that Proteobacteria play a key role in carbon, nitrogen, and sulfur cycling, supports oyster survival and growth and exhibits strong resistance to environmental extremes (Spain et al., 2009; Asmani et al., 2016; Bernal et al., 2017; Delmont et al., 2018). As to the genus level, *Vibrio* was the most prevalent and abundant genus, which has also been found in other studies (Lei et al., 2025). The absolute concentration of oysters from this region ranged from 10^7^ ~ 10^9^ with an average of 10^8^ copies/g (equivalent of CFU), which accounted for more than 90% of the total bacteria in certain oyster samples. This bacterial load level was approximately equivalent to total human fecal bacteria (10^8^ ~ 10^11^ copies/g) and comparable to that of soil (10^8^ ~ 10^9^ copies/g) (Zhang et al., 2017; Tkacz et al., 2018), suggesting their high abundance in oysters. However, due to the limited taxonomic resolution of the V3–V4 sequence of 16S rRNA at the species level, we could not distinguish whether these oysters harbored pathogenic *Vibrio* species, such as *Vibrio cholerae*, *Vibrio parahaemolyticus*, and *Vibrio vulnificus* that are harmful to humans. Nevertheless, heightened attention is still needed regarding the proliferation and transmission of oyster-borne *Vibrio*, both within oyster farming regions and in the human population. This is particularly relevant in Yueqing Bay, which is reported as one of the regions with the most cases *of Vibrio vulnificus* infection (Zhongqiu Lu et al., 2003; Lu, 2025), and oysters are also identified as one of the most reported *Vibrio* carrier marine species (Daniels, 2011; Froelich et al., 2013; Baker-Austin et al., 2018).

The bacteriome of oysters from Yueqing Bay exhibits a spatially distributed pattern. The structure of the oyster bacteriome differed among the four sampling sites of Yueqing Bay, which was supported by their *α* diversity (including community evenness and richness) and *β* diversity. Such spatial variation in bacteriome distribution is attributed to the heterogeneity of environmental factors, which is in turn driven by the geographic location. This observation is consistent with previous studies (Wan et al., 2021; Li et al., 2025). Notably, salinity emerged as the most influential factor shaping the bacterial communities of oysters. Given the high abundance of *Vibrio* in these oysters, the influence of salinity on the bacterial community is likely mediated through its impact on *Vibrio*, as confirmed by the positive correlation between salinity and *Vibrio*. As we know, oysters are typically farmed under optimal salinity conditions (Funo et al., 2015). Moreover, most *Vibrio* are halophilic bacteria that inhabit aquatic environments. Through filtering, *Vibrio* are transferred from water to oysters, establishing an association between the two. Even minor fluctuations in salinity can affect both the oysters themselves and their associated *Vibrio* symbionts, thereby changing the composition of the oyster bacteriome. This finding has been validated by previous studies (Lei et al., 2025). Furthermore, the optimal salinity of *Vibrio* growth is reported to be 20 ppt (Lei et al., 2025). Among the four sampling sites, D4 had the most suitable salinity for *Vibrio* proliferation, which explains why *Vibrio* was identified as a marker taxon in this site via LDA analysis. In addition to salinity, other environmental factors also exerted a significant effect on the bacterial community, such as TDS, DO, NO_3_^−^-N, and NH_4_^+^-N. Their influences are likely linked to specific genera such as *Pseudoalteromonas* and *Citrobacter*. These genera are known to be actively involved in biogeochemical cycling, such as C, N, and S, and contribute to oyster survival and growth (Dubilier et al., 2008; Fiore et al., 2010). For example, *Pseudoalteromonas* plays a key role in alginate degradation, which is an important marine organism carbon source (Xu et al., 2021). Additionally, as salinity-tolerant bacteria, these bacteria are also efficient protease producers that play important ecological roles in nitrogen degradation (Chen et al., 2020). Notably, site D5 exhibited the lowest NH4^+^-N and TN, which were significantly correlated with the enrichment of *Pseudoalteromonas* at this site (supported by LEfSe analysis). These results suggested that the influence of environmental factors on specific genera is determined by their functional potential, which ultimately shapes the oyster bacteriome structure. Taken together, our study found that geographically driven environmental heterogeneity influences the abundance of specific bacterial taxa in oyster, thereby altering the composition and structure of their bacteriomes. However, the underlying mechanisms, especially the specific response of bacterial species to environmental factors require further investigation.

Stochastic processes shape the oyster-associated bacteriome. The joint effect of deterministic and stochastic processes on microbial community assembly is widely recognized in the field of microbial ecology, but their relative importance varies by environments (Caruso et al., 2011; Wan et al., 2021; Riddley et al., 2025). For example, in fresh water lake, deterministic processes determine the bacterioplankton assembly (Wan et al., 2021), while in an estuary, stochastic processes appear to be much more important than deterministic processes (Li et al., 2025). In our study, we found that the bacteriome of the oyster is dominated by stochastic processes. This indicated that ecological drift exerts a strong influence on oyster bacterial community composition. A potential explanation for this is that the bacteriome in oysters is derived from filtering water in the surrounding environments, where bacterial dispersal is passively driven by water hydrodynamic forces. This intense hydrologic mixing can drive strong ecological drift with microbial communities (Bai et al., 2020). Indeed, oyster-associated bacteriome composition is highly reliant on the bacterial communities in the surrounding environment—a pattern consistent with previous findings that the host’s habitat is a key determinant of the microbiome in fishes (Kim et al., 2021). Therefore, the oyster-associated bacteriome was strongly impacted by a stochastic process. Furthermore, increased ecological drift may lead to a higher turnover rate, which is hypothesized to promote higher community diversity. This aligns with our observation that oysters from site D5, where the stochastic process exhibited a stronger influence, show high diversity (community richness). Oyster at this site also exhibited a higher number of marker bacteria compared to others (Figure 2, LEfSe). Additionally, although we identified that spatial difference determined the environmental heterogeneity that influences the structure of the oyster bacteriome, the geographic distance of these four sampling sites is still small (their altitude difference ranged within 0.0309°N and longitude difference ranged with 0.0122°E). Over the long term, therefore, minor fluctuations in environmental factors may thus only impact a limited subset of bacterial species, a pattern driven by inherent differences in the local microenvironment and the functional redundancy of bacterial communities in responding to environmental perturbations. For example, we found salinity significantly correlated with *Pseudoalteromonas* and *Vibrio*, while these genera contain numerous species capable of responding to environmental changes. Therefore, we consider that despite environmental factors influencing specific bacterial abundance, this may not alter long-term ecological processes, where higher biodiversity enables compensatory responses, where declines in one species due to unfavorable conditions are offset by another functionally similar species, which have been reported before (Fuhrman et al., 2015; Firkowski et al., 2022). Additionally, from the host perspective, a stable bacteriome is critical for oyster survival, and a stochastic process may represent an effective way for maintaining this stability. Specifically, ecological drift causes random fluctuations in species abundance, thereby allowing the bacteriome to act as buffer pools that generate substantial community diversity and resilience to environmental perturbations (Loreau et al., 2021; Firkowski et al., 2022; Li et al., 2025). To confirm these findings, further studies should include more samples from large geographic distance and measure a broader range of environmental factors related to bacterial growth.

## 5 Conclusion

In summary, our study found that geographic location drives variations in environmental factors, which in turn determine the structure of the oyster bacteriome. Notably, the assembly of the oyster bacterial community is dependent on stochastic processes. These findings provide fundamental insights into the pan-bacteriome of oysters from typical farming regions in Yueqing, China, while clarifying the relationship between environmental factors and the oyster bacterial community and the assembly process of the bacterial community. Such insights deepen our understanding of how the environmental conditions influence oyster farming, not only supporting the targeted optimization of oyster production but also laying a foundation for proactive management of oyster and public health. For instance, by adjusting the salinity level, we can explore strategies to regulate the oyster bacteriome to suppress the proliferation of pathogenic *Vibrio* and reduce their associated infection risks. Finally, continuous monitoring of the oyster-associated bacteriome is recommended, as it would help illuminate responses to environmental changes (e.g., climate change) and provide early warnings for environmental pollution.

## Data Availability

The datasets presented in this study can be found in online repositories. The names of the repository/repositories and accession number(s) can be found in the article/Supplementary material.
