# Supplementary material for: Insights into community profiles, environmental influence, and assembly mechanisms of oyster-associated bacteriome from Yueqing Bay, China via absolute quantitation by metabarcoding
Source: Front Microbiol. 2026 Apr 10;17:1752237. doi: 10.3389/fmicb.2026.1752237 (PMC13108226; doi:10.3389/fmicb.2026.1752237)
Supplement: Supplementary file 4 [file Table_4.docx]

**Supplementary Information**

**Insights into Community Profiles, Environmental Influence and Assembly Mechanisms of Oysters Associated Bacteriome from Yueqing Bay, China via Absolute Quantitation by Metabarcoding**

Huai Lin ^1^, Xin Li ^1^, Linhao Chen ^1^, Tingting Zhang^1^, Fengxia Yang ^2^, Yi Luo ^1*^

^1^State Key Laboratory of Water Pollution Control and Green Resource Recycling, School of the Environment, Nanjing University, Nanjing, China.

^2^Agro-Environment Protection Institute, Ministry of Agriculture and Rural Affairs, Tianjin, China


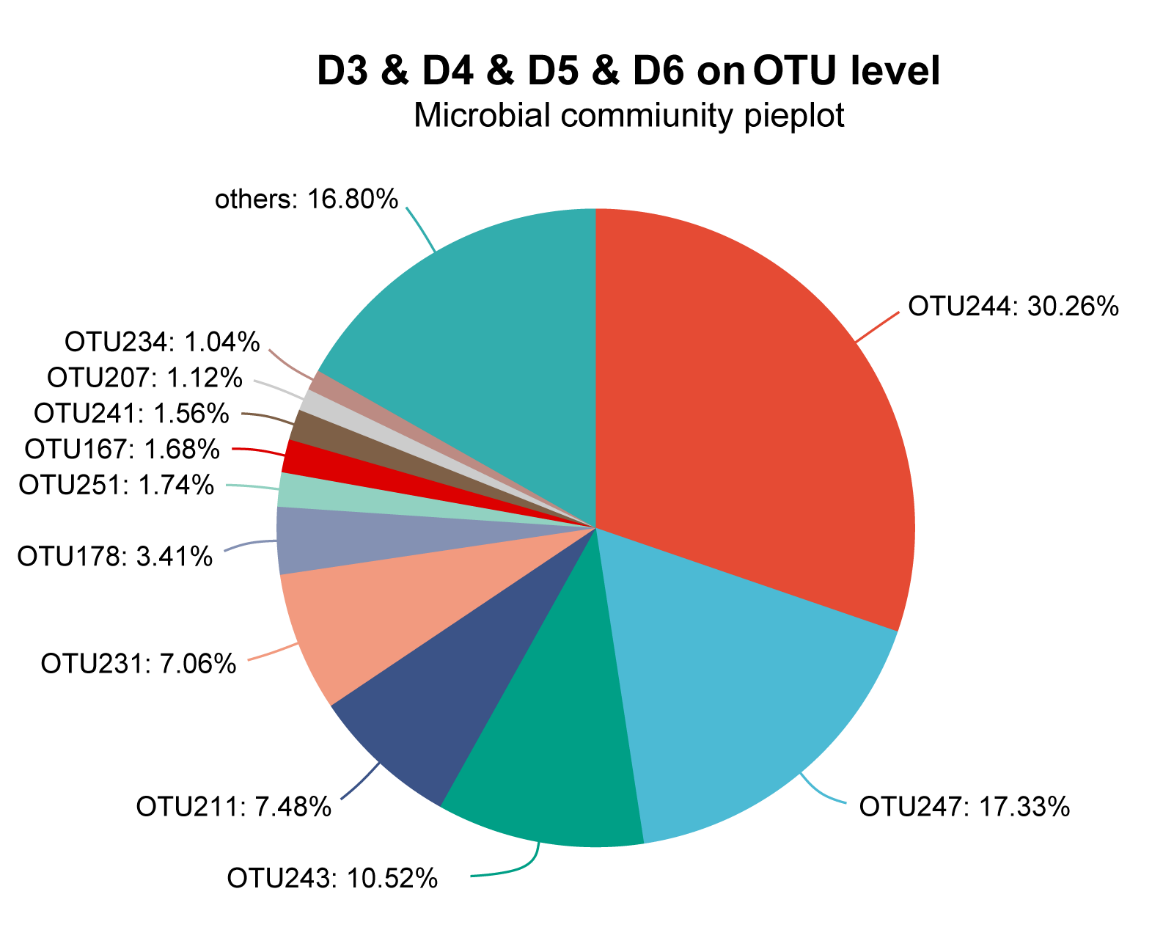


**Figure. S1** Top 10 co-shared OTU of different sampling sites


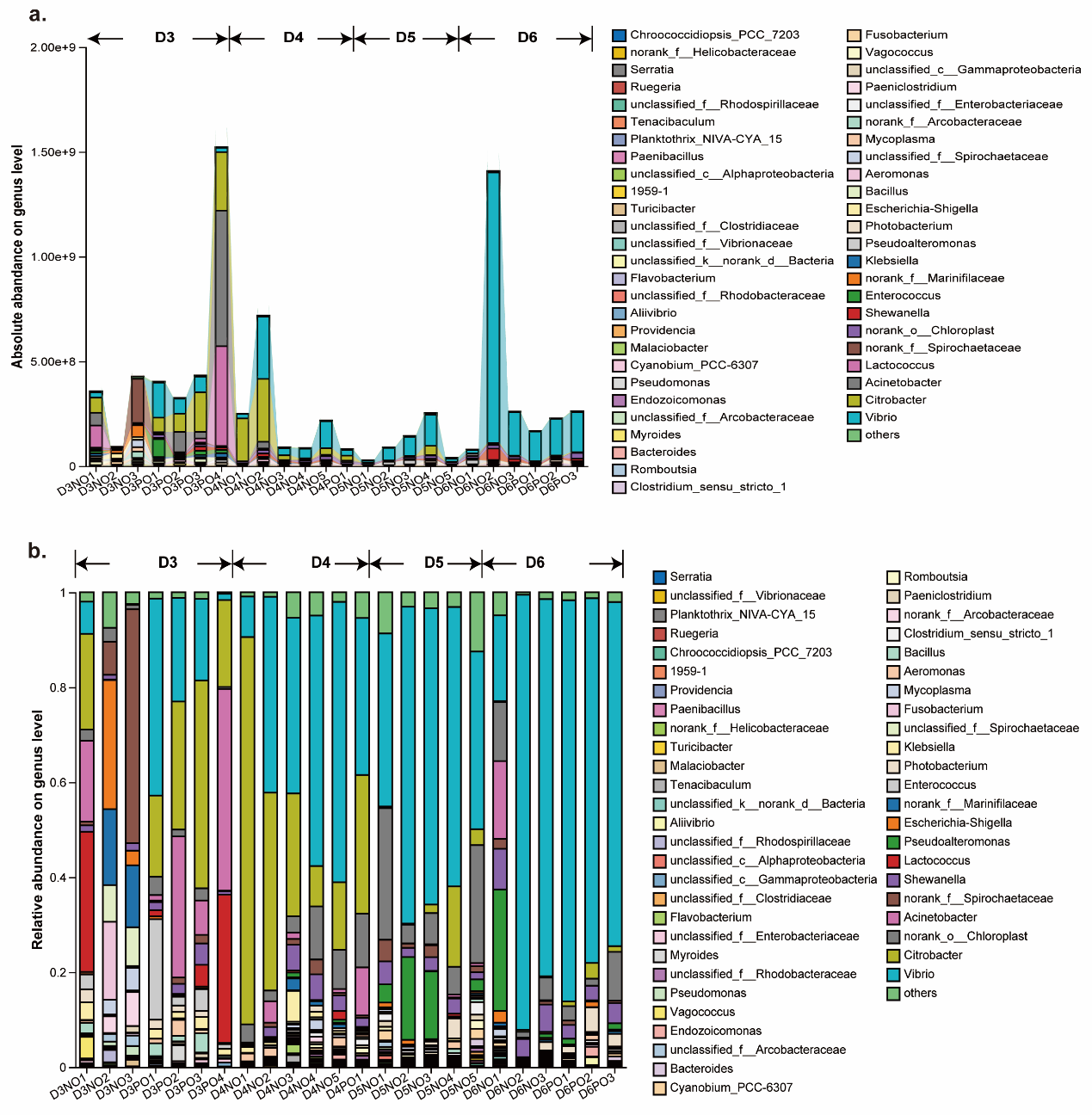


**Figure. S2** Absolute (a) and relative (b) abundance of bacteria at genus level among different sites.


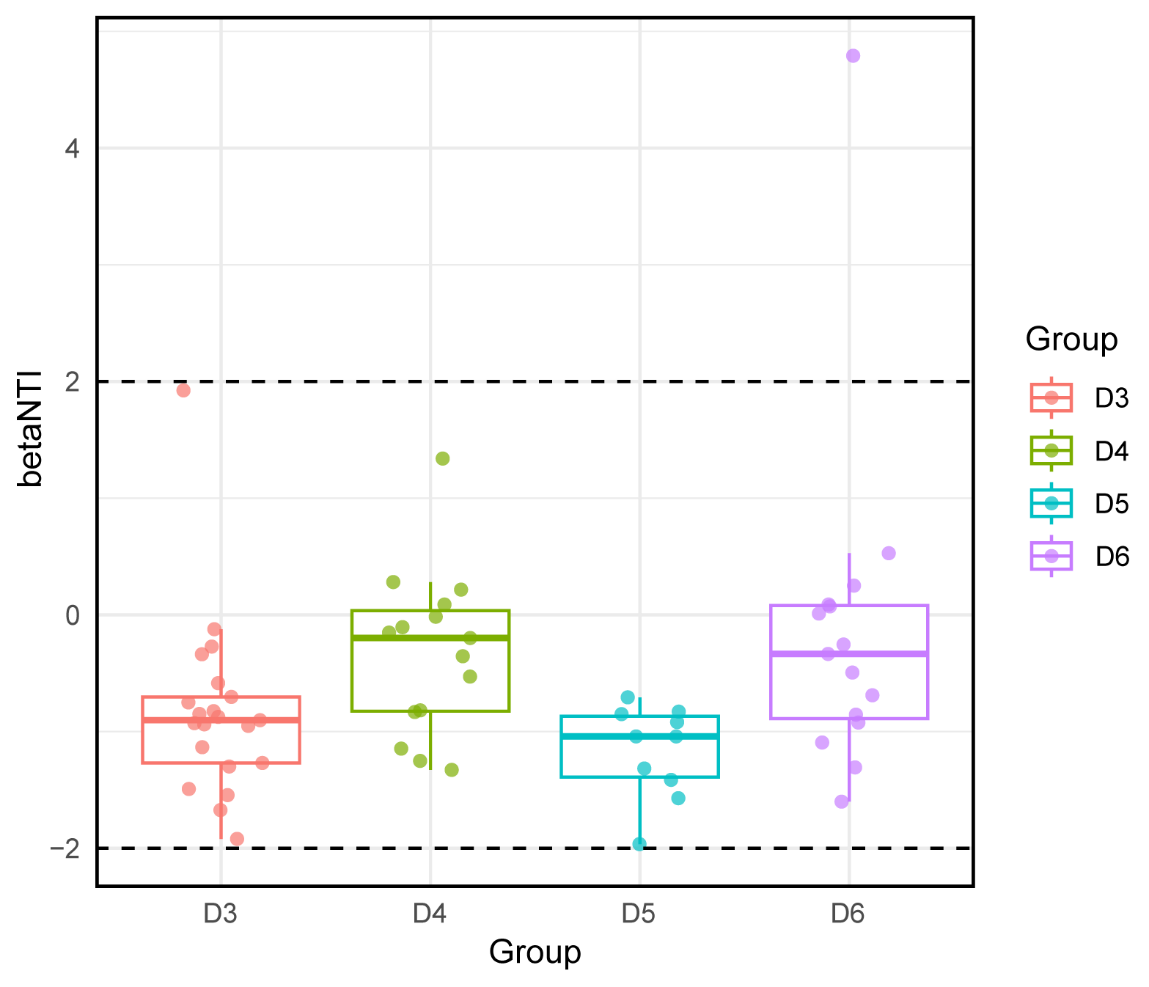


**Figure. S3** βNTI of oyster bacterial communities from different sites
